# Mechanical and Strain-Sensing Capabilities of Carbon Nanotube Reinforced Composites by Digital Light Processing 3D Printing Technology

**DOI:** 10.3390/polym12040975

**Published:** 2020-04-22

**Authors:** Alejandro Cortés, Xoan F. Sánchez-Romate, Alberto Jiménez-Suárez, Mónica Campo, Alejandro Ureña, Silvia G. Prolongo

**Affiliations:** Materials Science and Engineering Area, Escuela Superior de Ciencias Experimentales y Tecnología, Universidad Rey Juan Carlos, C/ Tulipán s/n, 28933 Móstoles, Madrid, Spain; xoan.fernandez.sanchezromate@urjc.es (X.F.S.-R.); alberto.jimenez.suarez@urjc.es (A.J.-S.); monica.campo@urjc.es (M.C.); alejandro.urena@urjc.es (A.U.); silvia.gonzalez@urjc.es (S.G.P.)

**Keywords:** additive manufacturing, 3D printing, digital light processing, DLP, structural health monitoring, thermoset, nanocomposites, carbon nanotubes, CNTs

## Abstract

Mechanical and strain sensing capabilities of carbon nanotube (CNT) reinforced composites manufactured by digital light processing (DLP) 3D printing technology have been studied. Both CNT content and a post-curing treatment effects have been analyzed. It has been observed that post-curing treatment has a significant influence on mechanical properties, with an increase of Young’s modulus and glass transition temperature whereas their effect in electrical properties is not so important. Furthermore, the strain sensing tests show a linear response of electrical resistance with applied strain, with higher values of sensitivity when decreasing CNT content due to a higher interparticle distance. Moreover, the electrical sensitivity of bending tests is significantly lower than in tensile ones due to the compression subjected face effect. Therefore, the good gauge factor values (around 2–3) and the high linear response proves the applicability of the proposed nanocomposites in structural health monitoring applications.

## 1. Introduction

In recent years, 3D printing is positioning as a competitive manufacturing technology with respect to traditional manufacturing technologies [1]. The main reasons that make this possible are, among others, the massive customization capability of the final product, as well as the chance of making small batch sizes without increasing costs derived from the manufacturing process. Moreover, it allows manufacturing components with almost total freedom in the design, thus being able to obtain high complexity parts, which are impossible to perform using any of the traditional manufacturing technologies. In addition, it makes possible to shorten supply chain links and lead times, reducing assembly, storage, and transportation costs [2,3,4,5].

However, 3D printing technologies are fully developing, so there is still a wide field for improvement in both the production processes and the materials that can be used [6]. In this context, developing materials with new functionalities or enhanced properties has acquired significant relevance [7]. In particular, carbon nanotube (CNT) doped resins has been the subject of numerous studies in the last decades due to their great mechanical, thermal, and electrical properties [8,9,10]. Their addition in low contents into an insulator resin allows the formation of electrical percolating networks inside the material, leading to an increase in electrical conductivity of the material of several orders of magnitude [11].

One of the main applications of these materials is structural health monitoring (SHM), which consists in a real-time evaluation of the structure integrity, being able to detect, locate, and quantify both strain and damage, and even make a remaining useful life prognosis [12]. In this context, CNT doped nanocomposites have a great potential and applicability. This is explained by the intrinsic piezoresistivity of CNTs [13,14], the contact conductive mechanism between CNTs and the tunneling effect that takes place between adjacent nanotubes. The combination of these factors leads to an enhanced strain sensitivity, much higher than those achieved for conventional metallic gauges [15,16,17,18,19].

There are several studies of materials manufactured by 3D printing with CNTs as conductive fillers. Some specific examples are described below. Using the fused deposition modeling (FDM) technique, Kürçad et al., developed 3D printed parts with enhanced mechanical and electrical properties [20], Josef F. Christ et al., studied bidirectional and stretchable piezoresistive sensors [21], Kyuyoung et al., manufactured multiaxial force sensors [22] and Dong Xiang et al., obtained highly elastic strain sensors with an outstanding strain-sensing performance [23]. Regarding direct write (DW) 3D printing technique, Kambiz Chizari et al., developed highly conductive nanocomposites for electromagnetic interference shielding applications [24], Shin et al., researched about bioactive 3D printed flexible electronics [25] and Farahani et al., explored the strain sensing capabilities of CNT doped composites combining the direct write technique with UV curable formulations [26]. Besides that, Gustavo Gonzalez et al., settled the bases of manufacturing CNT doped materials with a commercial digital light processing (DLP) 3D printer [27]. Using this manufacturing technique, Tiller et al., developed a piezoelectric microphone combining different materials [28] and Mu et al., explored some applications like hollow capacitive sensors and smart structures with shape memory effects [29]. Despite this, there is still a wide field of research about photocurable inks for SHM purposes.

The present study deals with the development of a conductive ink for DLP 3D printing technology with self-sensing capabilities based on a commercial photocurable resin doped with CNTs. First, CNT content was varied in order to analyze its influence on electrical properties of nanocomposites. In this regard, the electrical percolation threshold has been achieved. Then, strain-sensing capabilities have been deeply explored at tensile and bending load conditions, in order to better determine the influence of load state and post-curing treatment on the sensitivity of these materials, which allows selecting the best combination of mechanical and electrical properties as a function of the desired application.

## 2. Materials and Methods

Different dispersions were prepared with different CNT contents (0.030, 0.050, 0.075, 0.100, and 0.150 wt %) in order to obtain the electrical percolation threshold of the nanocomposite. Moreover, strain sensitivity and mechanical properties were measured by tensile and three-point bending tests.

In addition, TOM and FEG-SEM characterization was performed to study the dispersion state as well as its stability. Finally, DSC tests were carried out to know the curing degree of the manufactured nanocomposites.

### 2.1. Materials

CNTs used in this study were the NC7000 supplied by Nanocyl (Sambreville, Belgium). These multi-walled carbon nanotubes (MWCNTs) have an average diameter of 9.5 nm and a length up to 1.5 μm. They also present high UV resistance, which is important to prevent properties loss during manufacturing process.

High Temp Resin V1, supplied by Formlabs (Somerville, MA, USA), was used as matrix. It is an acrylate-based UV-curable resin designed to be used in high temperature applications since it performs a heat deflection temperature under 0.45 MPa of 298 °C.

### 2.2. Manufacturing of the Nanocomposite Specimens

First, CNTs were dispersed in the resin by three roll milling technique (Exakt 80E by Exakt Technologies, Oklahoma City, OK, USA), performing a seven cycles process, optimized in previous studies [30]. Table 1 shows the parameters for each cycle, where the speed of the last roll is kept constant at 250 r.p.m.

Once the dispersion is completed, the mixture was added to the resin vat of the 3D printer used (B9Creator by B9Creations, Rapid City, SD, USA) for the manufacturing of the different test specimens. The printing technique is based on digital light processing (DLP) technology, in which the light source of a digital projector is focused on the bottom surface of the resin vat with the desired geometrical shape, photocuring the first layer of the specimen. This process is repeated layer by layer until the complete part is finished. The most relevant printing parameters were 30 µm of layer thickness and 5.12 s of exposure time per layer, except for those specimens with a 0.150 wt % CNTs, where the exposure time was increased to 6.84 s. This is due to the higher CNT content that induces a more prevalent UV light shielding effect caused by CNTs, reducing UV radiation exposure of the photoinitiator and leading thus to an underexposure condition. On the other hand, overexposure conditions were observed for specimens with CNT contents below 0.100 wt % and longer UV light exposure times than 5.12 s.

Six specimens for each CNT content (0.030, 0.050, 0.075, 0.100, and 0.150 wt %) and test type (electrical conductivity, tensile, and three-point bending tests) were 3D printed. Dimensions were established according to the ASTM D257, ASTM D638, and ASTM D790 standards respectively.

Finally, half of the specimens of each manufacturing condition underwent an UV post-curing treatment during 30 min in order to study the influence of this post-curing stage on the strain sensing capability and the mechanical properties. This stage was carried out in a B9A-LCB-020 oven by B9C reations.

**Figure 1:**
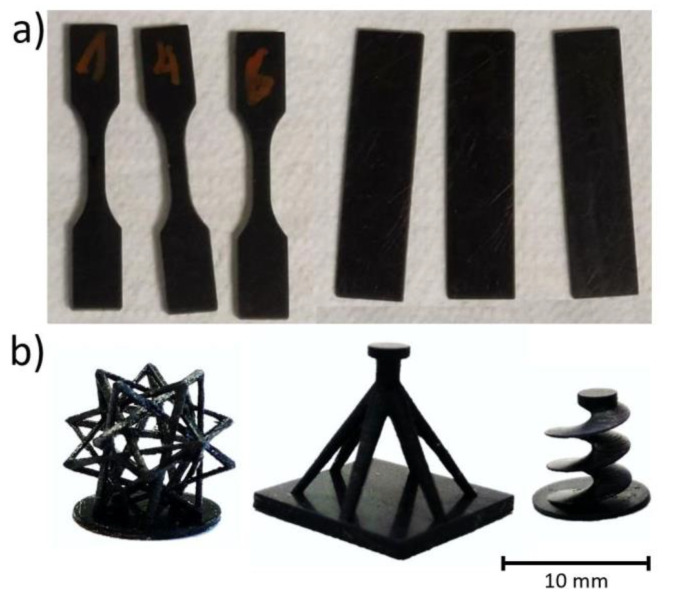
Examples of 3D printed parts with 0.100 wt % CNT. (**a**) Tensile and three-point bending test specimens and (**b**) complex geometry parts.

Figure 1 shows some examples of 3D printed parts with a 0.100 wt % CNT. First, manufactured tensile and three-point bending specimens are shown in Figure 1a while Figure 1b shows some parts with complex geometries, which demonstrates the cabability of printing conductive complex parts with this material and manufacturing technique.

### 2.3. Characterization

#### 2.3.1. TOM and FEG-SEM

Nanoparticle dispersion of uncured CNT-acrylate mixture, containing 0.100 wt % CNTs, was analysed by using a Leica transmission optical microscope (TOM) equipped with a Nikon 990 camera (Tokyo, Japan). In this regard, several samples were taken at different times after dispersion procedure in order to get a deeper knowledge about the stability of the dispersion. Moreover, an analysis of fracture surface under cryogenic conditions was carried out to better characterize the CNT dispersion in the final nanocomposite. These fracture surfaces were observed by a field emission gun SEM (FEG-SEM) using a Nova NanoSEM 230 apparatus from Philips (Amsterdam, Netherlands).

#### 2.3.2. DSC

**Figure 2:**
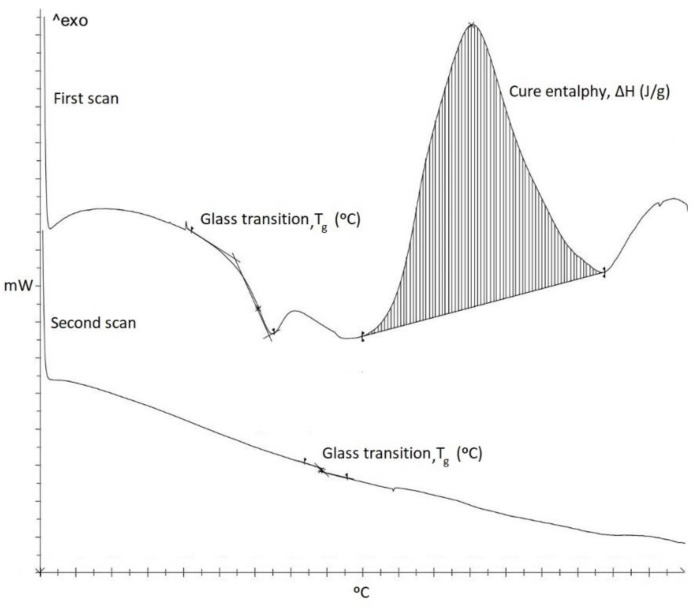
Example of DSC test including first and second scan.

Non-isothermal Differential Scanning Calorimetry (DSC) tests were carried out with a Mettler-Toledo 882e (Columbus City, OH, USA) device from 0 to 300 °C at 10 °C/min. Glass transition temperature (*T*_g_) and enthalpy (ΔH) were evaluated in order to compare the curing degree achieved between the different specimens. Despite the used resin is an UV curable one, it is possible to compare the curing degree between specimens since it also presents thermal post-curing capabilities. Thus, two scans were performed to ensure that all the thermal curing enthalpy was recorded during the first scan. Figure 2 shows an example of DSC test including first and second scan. In the first scan, *T*_g_ and normalized ΔH per gram of specimen are obtained. In addition, the second scan confirms that the thermal curing process was completed in the first scan and then all thermal ΔH was recorded.

DSC samples were taken from the same corner of the 3D printed three-point bending test specimens of each manufacturing condition in order to obtain representative results, since the curing degree of the specimens may vary depending on the specimen type and zone.

#### 2.3.3. Electrical Conductivity

DC volume conductivity tests were performed in 10 × 10 × 1 mm^3^ samples to characterize their electrical properties accordingly to ASTM D257 standard. It was determined by the slope of I–V curve with a voltage window of 0–50 V using a Source Measurement Unit (SMU, Keithley Instrument Inc. mod. 2410, Cleveland, OH, USA).

### 2.4. Electromechanical Tests

**Figure 3:**
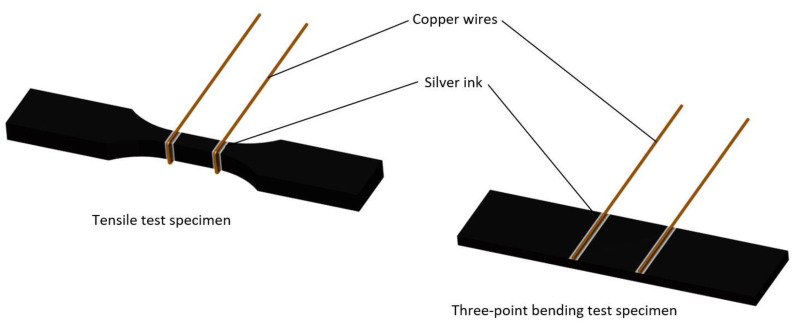
Schematic of electrode disposition for tensile and three-point bending test specimens.

Tensile and three-point bending tests were carried out in order to determine the electromechanical behavior of the manufactured materials. These tests were carried out in a Zwick Z100 (Ulm, Germany) universal tensile machine accordingly to standards ASTM D638 and D790, respectively. Tensile specimens were tested at a rate of 5 mm/min while bending tests were first conducted at 1 mm/min up to 0.7% strain to determine the flexural modulus and then at 10 mm/min up to failure to obtain the flexural strength. Simultaneously to mechanical tests, electrical characterization was done by using an Agilent 34410 A module (Santa Clara, CA, USA). It was conducted by means of electrical resistance measurements between two electrodes made of copper wire and silver ink in order to ensure a good electrical contact with sample surface. A schematic of electrode disposition is shown in Figure 3. It is important to highlight that, for the tensile test specimens, the electrodes are positioned all around the perimeter of the cross section while for three-point bending ones, the electrodes are positioned just on the tensile face.

Here, the electrical sensitivity to the applied strain, or gauge factor, is estimated as the change of the normalized resistance ($\Delta R/R_{0}$) divided by the applied strain (ε). The electrical sensitivity was calculated at low strain levels, up to 0.01 mm/mm.

## 3. Results and Discussion

### 3.1. Dispersion State Analysis

**Figure 4:**
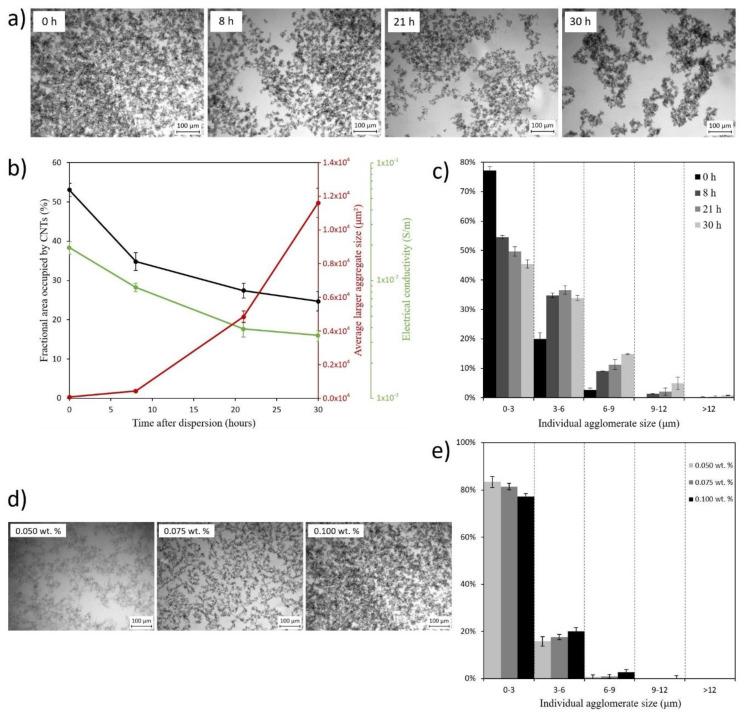
Changes on the dispersion state as a function of time after dispersion process was performed and as a function of CNT content. (**a**) TOM micrographs of dispersion containing 0.100 wt % CNT at 0, 8, 21, and 30 h after dispersion was carried out; (**b**) fractional area occupied by CNTs, average larger aggregate size and their influence in electrical conductivity as a function of time since after dispersion; (**c**) individual agglomerate size as a function of time after dispersion; (**d**) TOM micrographs at 0 h after dispersion as a function of CNT content; and (**e**) individual agglomerate size at 0 h after dispersion as a function of CNT content.

Figure 4a–c show the changes in the dispersion state as a function of time after dispersion was performed and how it affects the electrical conductivity of manufactured nanocomposites. Individual CNT agglomerates and larger CNT aggregates can be distinguished which refer to the aggregation of multiple individual CNT agglomerates. TOM micrographs in Figure 4a evince the worsening of dispersion state from a uniform CNT distribution to a re-aggregated one along time, which are quantified in both Figure 4b,c. Firstly, Figure 4b reveals that the fractional area occupied by CNTs decreases about a 57% over time while larger CNT aggregates grow in size more than a 500%. It means that there is a CNT re-agglomeration over time, as can be also distinguished in the histogram of Figure 4c. All these phenomena are closely linked because of the van der Waals interacting forces affecting CNTs that induce a re-agglomeration effect for both individual CNTs and aggregates. This could consequently affect the electrical and mechanical properties, among others. In this case, by analyzing the changes in electrical conductivity, a decrease of almost one order of magnitude was observed for samples taken 20 h after dispersion was performed (Figure 4b). It is explained since the more aggregated the CNTs are, the more difficult the electrical current flow through the composite is, as the CNTs tend to cluster into isolated colonies, making the electrical resistance by tunneling effect among the different colonies much higher. Finally, electrical conductivity tends to stabilize from 21 h onwards.

These results demonstrate the importance of additive manufacturing with less time delay since the nanoparticles are dispersed in the resin, to prevent loss of properties in the resulting nanocomposite as this time delay induces a re-agglomeration of nanoparticles which would lead to a decrease of electrical conductivity as well as the presence of higher aggregates that could affect mechanical properties.

Moreover, the effect of CNT content can be observed in the TOM images of Figure 4d. It can be noticed that the higher the amount of CNTs the higher the presence of aggregates in the mixture. This can be confirmed by an analysis of the aggregate size (Figure 4e) where the correlation between the larger aggregates and the smaller ones increases with CNT content due to a higher tendency of the CNTs to be agglomerated.

**Figure 5:**
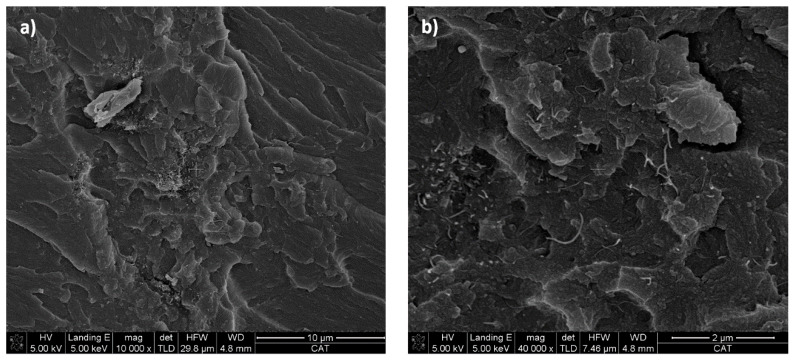
FEG-SEM micrographs showing CNTs distribution of the 0.100 wt % CNT specimen at (**a**) low magnifications and (**b**) high magnifications.

Regarding 3D printed composites, Figure 5 shows FEG-SEM micrographs of a specimen with 0.100 wt % CNTs. At low magnifications, Figure 5a, a homogeneous distribution of CNT can be seen. Despite this, it can be distinguished that there are still some aggregates of nanoparticles, since the calendering process cannot completely disperse the CNT in the resin, being less effective than in other systems with higher viscosity [31]. However, at higher magnifications, Figure 5b an adequate distribution of nanoreinforcement is observed. In addition, CNTs appear to be well integrated in the matrix, which is important to ensure adequate mechanical behavior [32].

### 3.2. Electrical Conductivity

**Figure 6:**
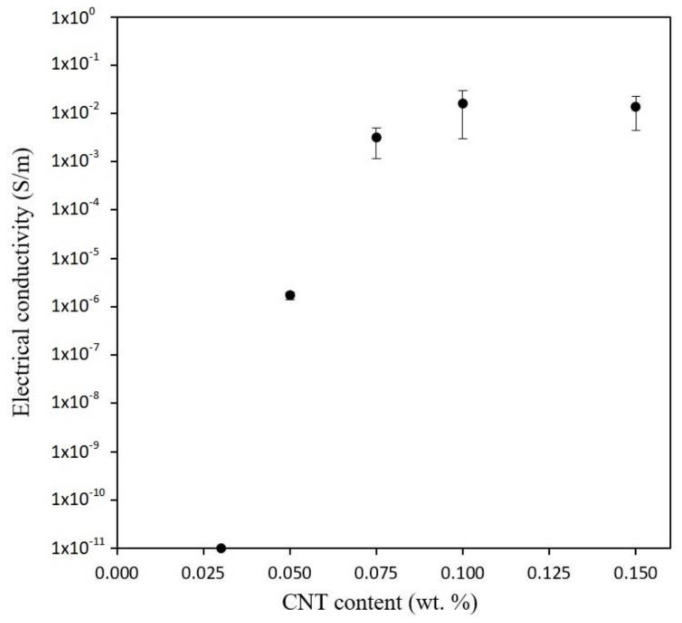
Electrical conductivity of 3D Printed specimens as a function of CNT content.

Figure 6 shows the electrical conductivities determined in 3D printed specimens for the different tested conditions. Here, electrical percolation threshold was determined as the critical CNT content where the material becomes electrically conductive, which corresponds to the moment where a sudden increase of electrical conductivity was observed. In this case, it is found at 0.050 wt %, which is in good agreement to that found in other studies with similar CNTs [31].

For contents above percolation threshold, up to 0.100 wt %, an increase of electrical conductivity was observed as expected due to a higher volume fraction of nanoparticles. However, conductivity decreases at a content of 0.150 wt %. The reason of the detriment on electrical properties was found in the role of CNT agglomerates inside the material. Up to CNT contents below 0.100 wt %, a relatively good CNT dispersion was found, but, at higher contents, a supersaturation of CNTs in the percolation network takes place, which implies a higher tendency to form aggregates. These aggregates create preferential electrical pathways but also regions without enough nanoreinforcements—called non-percolated regions—that do not form effective conductive pathways.

**Figure 7:**
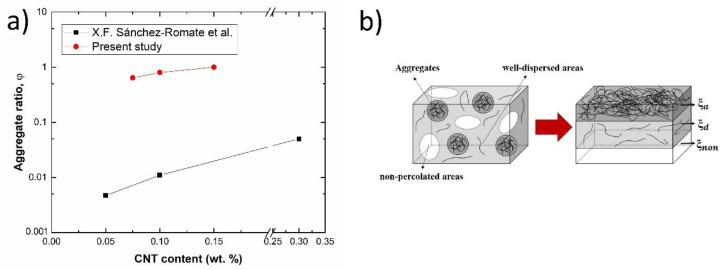
(**a**) Values of aggregate ratio as a function of CNT content and comparison to a previous study [31] and (**b**) schematics of block disposition for the electrical model indicating the aggregated, well-dispersed and non-percolated regions [31] (Reproduced with permission from X.F. Sánchez-Romate et al., Composites Science and Technology; published by Elsevier, 2019).

More specifically, an analysis of CNT aggregation state for the different conditions is summarized in Figure 7a. Here, the role of agglomerated and dispersed areas is highlighted. It was based on a previously proposed analytical model [31], whose basic principle is shown in the schematic of Figure 7b. The material can be divided in different regions accordingly to the aggregation state of CNTs within the network, where *ξ_a_*, *ξ_d_*, and *ξ_non_* correspond to the fraction of agglomerates, well dispersed and non-percolated regions, respectively. Thus, electrical conductivity can be calculated by the equivalent parallel circuit with the following Formula (1)

(1) $$\frac{1}{R}=\xi_{a}\cdot \frac{1}{R_{a}}+\xi_{d}\cdot \frac{1}{R_{d}}+\underset{\sim 0}{\underset{︸}{\xi_{non}\cdot \frac{1}{R_{\infty }}}}\to R=\frac{R_{a}R_{d}}{\left(\xi_{d}R_{a}+\xi_{a}R_{d}\right)}$$

In this case, and knowing the CNT geometry, it is possible to estimate the values of *ξ_a_*, *ξ_d_*, and *ξ_non_* for each nanoparticle content as well as to calculate the correlation between aggregated and well dispersed areas, also called aggregate ratio $\varphi =\xi_{a}/\xi_{d}$. It can be observed that the higher CNT content the higher the aggregate ratio, which means a poor dispersion of nanoparticles. When analyzing in detail, it is observed that the values of aggregate ratio are much higher than those found for similar contents in the previous study. This is explained because of the lower viscosity of the resin used in this research in comparison to that used in the previous study (600 and 5600 mPa·s, respectively). This leads to a lower effectiveness of the three roll milling process due to a reduction of the shear forces involved during the dispersion procedure, also explaining the lower conductivity values obtained in comparison to other studies [31]. Furthermore, it can be also observed that the aggregate ratio increases with CNT content. It indicates that the correlation of larger aggregates to well-dispersed areas is increasing, as previously observed by the analysis of aggregate size of Figure 4e,f with an increase of larger aggregates with CNT content. It is important to point out that this model only gives information about the ratio of aggregate to well-dispersed areas but does not give information about the values of each individual fraction.

However, the percolation threshold obtained in this study is lower than found in other studies based on CNT doped resins for DLP 3D printing technology, which suggests a better dispersion of CNTs than those obtained in these studies with similar [27] and even higher aspect ratios of the nanofillers [29].

### 3.3. Mechanical Properties

**Figure 8:**
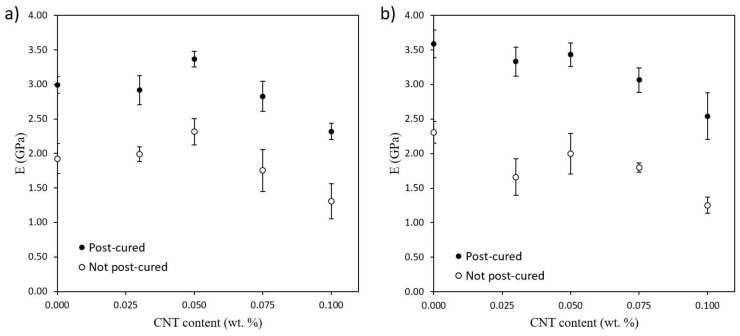
Young’s modulus of 3D printed specimens as a function of CNT content. (**a**) Tensile tests and (**b**) three-point bending tests.

Mechanical properties were measured by tensile and three-point bending tests. Figure 8 shows Young’s modulus of 3D printed specimens with and without UV post-curing treatment as a function of CNT content. UV post-cured specimens present a higher stiffness in both tensile (Figure 8a), and three-point bending tests (Figure 8b), compared to the not post-cured ones. This is because of the higher crosslinking degree reached during post-curing treatment, limiting polymeric chains movement leading, thus, to a strengthening of the material [33]. However, the effect of the CNT content is quite more complex. On the one hand, there is an increase in tensile modulus at lower CNT contents due to the effective reinforcement of the nanotubes. On the other hand, there is a reduction of the Young’s modulus at higher CNT contents for both tensile and three-point bending tests, which could be explained by the higher UV light shielding effect caused by CNTs. They are black-colored, so they absorb part of the UV radiation from the 3D printer projector, blocking UV absorption by the photoinitiator, which leads to a lower curing degree and therefore to worse mechanical properties. This effect has already been observed by other authors and it can be sorted out by increasing exposure time per layer during the printing process, increasing the UV light intensity, adding higher photoinitiator concentrations or optimizing the post-curing stage [27,29,34]. Moreover, a higher content of nanoparticles could hamper the mobility of the chains during the photocuring process, leading to a lower curing degree and then to a worsening of mechanical properties [35].

**Figure 9:**
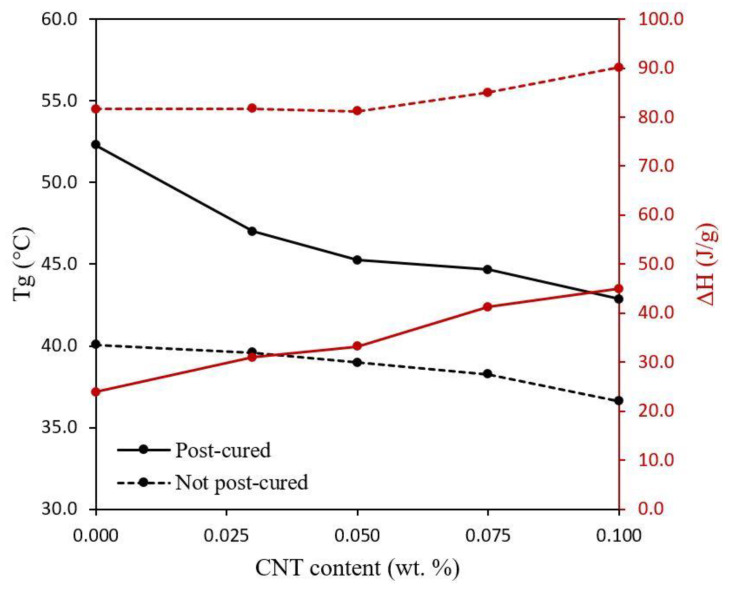
Glass transition temperature (*T*_g_) and cure enthalpy (ΔH) measured by DSC of 3D printed composites as a function of CNT content.

Both the effect of the UV post-curing treatment and the addition of CNTs on the mechanical properties above mentioned can be deeply explained through the performed DSC characterization, whose results are shown in Figure 9. Here, the post-cured specimens present a higher *T*_g_ than the not post-cured ones. This is due to the higher crosslinking degree achieved during UV post-curing treatment, improving the mechanical performance. Furthermore, the changes observed in *T*_g_ are directly related to ΔH measurements. The lower the ΔH, the higher the curing degree since it releases less energy correlated to the curing of the specimen during the DSC test.

Besides that, a decrease of the *T*_g_ when the CNT content increases is observed for both post-cured and not post-cured specimens. This is again explained by the lower curing degree achieved when increasing the CNT content because of the prevalence of the UV shielding effect, leading to a detriment of mechanical properties. This can be also confirmed by ΔH measurements, which raises with the increase in the content of CNTs. In addition, the changes in the *T*_g_ and ΔH, correlated to the curing degree, with the CNT content is more pronounced for the post-cured specimens. The UV post-curing treatment is performed once the specimen is printed so that the whole specimen is irradiated at once in the UV oven. Because of the UV shielding effect caused by CNTs, this post-curing treatment is much more effective on the outer regions of the specimen since the inner regions did not receive the same UV light intensity. Therefore, the post-cured specimens without CNTs show a much higher *T*_g_ than the specimens containing nanoreinforcements. This is because the neat resin specimen is clear-colored, without carbon nanoparticles absorbing part of the UV light from the UV oven so that both the outer and the inner regions can achieve practically the same crosslinking degree. Conversely, the *T*_g_ and ΔH of the as-fabricated specimens without post-curing treatment has not undergone such significant changes since the only UV light they have received comes from the 3D printer and it is emitted layer by layer, being that the UV shielding effect less pronounced.

Specimens containing 0.150 wt % CNTs has not been represented in both Figure 8 and Figure 9. The comparison of the mechanical properties and curing degree between specimens containing 0.150 wt % CNTs with respect to the other specimens is not particularly meaningful since they present different exposure times per layer, which changes their curing conditions. In addition, the significantly higher shield effect of the CNTs at these conditions did not make a complete curing possible, even with the UV-post curing and the samples presenting a very high distortion.

### 3.4. Structural Health Monitoring

**Figure 10:**
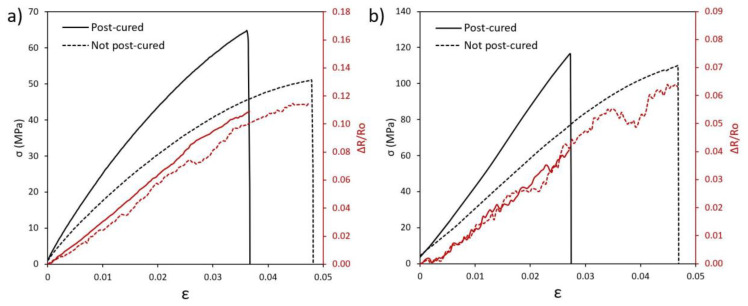
Examples of electromechanical tests comparing specimens with and without post-curing treatment in (**a**) tensile tests for specimens containing 0.100 wt % CNT and (**b**) three-point bending tests for specimens containing 0.050 wt % CNT.

Figure 10a shows an example of tensile electromechanical behavior for post and non-post cured specimens with 0.100 wt % CNT. As a first sight, the electromechanical properties are not significantly different between the post and not-postcured specimens. In fact, the variation of electrical resistance with applied strain is very similar, showing in both cases a mainly linear behavior. This indicates that the CNT network is not prevalently affected by the post-curing treatment as expected, since the gelation process takes places at the same time in both samples. Moreover, the linear behavior indicates a prevalence of in contact mechanisms over tunnelling effect ones, which changes in an exponential way with applied strain [36]. Bending tests (Figure 10b) show a similar electromechanical behavior with an increase of electrical resistance with strain, given by the increase of tunnelling distance between adjacent CNTs in the tensile-subjected face of the flexure specimen.

**Figure 11:**
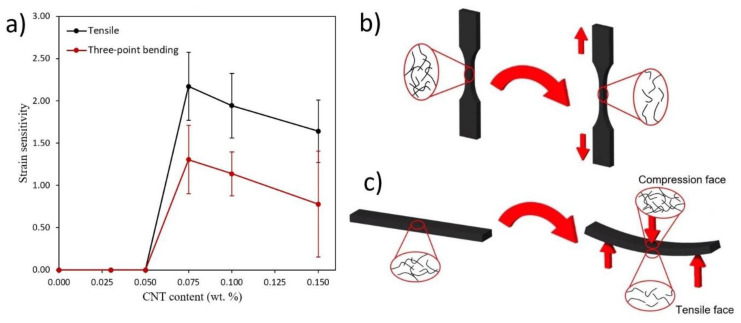
(**a**) Strain sensitivity as a function of CNT content for tensile and three-point bending tests, (**b**) schematic of CNTs distribution during tensile test, and (**c**) schematic of CNTs distribution during tensile test three-point bending test.

When comparing the strain sensitivity, defined by the change of the normalized resistance with applied strain, and summarized in Figure 11a, it is observed that there are slight differences attending the CNT content. On the one side, the highest values of sensitivity are obtained at the lowest nanoparticle content. This is explained by the previously commented prevalence of tunnelling mechanisms in the well-dispersed areas. This induces an exponential change of electrical properties and, thus, a higher variation of electrical resistance with mechanical strain. When increasing the CNT content, there is an increasing saturation of the CNT network, leading to a predominance of agglomerated areas. Here, the main conducting mechanisms are given by the electrical contact between adjacent CNTs, and the electrical resistance due to this effect is supposed to be invariable with applied strain. These statements are in good agreement with other studies [31,37,38] as well as with the calculated values of the aggregate ratio, previously shown in Figure 7a, indicating this prevalence of contact mechanisms through the aggregated areas when increasing the CNT content.

In addition, there is a decrease of electrical sensitivity when comparing tensile and bending tests. This is explained by the influence of compression-subjected face of the flexure specimen on the whole electrical resistance of the specimen, as observed in the schematics of Figure 11c. This compression-subjected face acts in an opposite way than the increasing electrical resistance due to tensile effects in the opposite face, leading thus to a reduction of sensitivity when comparing to pure-tensile loading state, shown in Figure 11b, as observed in other studies [39].

Therefore, the electromechanical results are robust and allows to better understand the role of the dispersion and main conducting mechanisms inside these materials.

## 4. Conclusions

The electrical and mechanical behavior of 3D printed CNT doped nanocomposites has been investigated for different CNT contents. In addition, the effect of an UV post-curing treatment has been also explored.

First, the dispersion state and stability were characterized by TOM and FEG-SEM, evincing the importance of 3D printing the doped resins within the first hours after the dispersion process was performed. A homogeneous CNT distribution was obtained for the specimens manufactured within the first hours after the dispersion process by calendering was completed. However, the low viscosity of the mixture favors the re-agglomeration effect between individual CNTs and aggregates. In addition, the proper CNT distribution achieved, allows obtaining a lower electrical percolation threshold, around 0.050 wt % CNTs, than the values compared to other studies with similar manufacturing techniques and materials.

Regarding mechanical properties, the UV post-curing treatment significantly increased material stiffness, determined by tensile and three-point bending tests due to the rise in the crosslinking degree of the polymeric chains. On the other hand, an increase of CNT content induces a decrease of Young’s modulus. This is explained because of the higher UV shielding effect, which leads to lower curing degrees and thus to worse mechanical properties.

Nevertheless, the best results in terms of strain sensitivity were also found for the lowest CNT contents since they are closer to the percolation threshold, with the tunnelling effect being the most dominant mechanism of electrical charge transport. Moreover, strain sensitivity was found to be significantly lower for three-point bending tests than for tensile tests as expected because of the effect of the compression-subjected face on the whole electrical resistance of the specimen.

Therefore, the results prove the excellent capabilities of CNT reinforced DLP-manufactured nanocomposites in strain-sensing applications and shed light into how an UV post-curing treatment and CNT content affects the electromechanical properties of these materials.

## Figures and Tables

**Table 1:** Gap distance between rolls during calendering process.

Gap 1 (µm)	Gap 2 (µm)	Number of Cycles
120	40	1
60	20	1
45	15	1
15	5	4
